# Back and neck pain: A comparison between acute and chronic pain–related Temporomandibular Disorders

**DOI:** 10.1080/24740527.2022.2067032

**Published:** 2022-07-01

**Authors:** Jack Botros, Mervyn Gornitsky, Firoozeh Samim, Zovinar der Khatchadourian, Ana Miriam Velly

**Affiliations:** a Department of Dentistry, Jewish General Hospital, Montreal, Quebec, Canada; b Faculty of Dental Medicine and Oral Health Sciences, Lady Davis Institute for Medical Research, Montreal, Quebec, Canada; c Faculty of Dentistry, McGill University, Montreal, Quebec, Canada; d Department of Dentistry, Montreal General Hospital, Montreal, Quebec, Canada; e Alan Edwards Pain Management Unit, Montreal General Hospital, Montreal, Quebec, Canada

**Keywords:** temporomandibular disorder, chronic pain, acute pain, neck pain, back pain, comorbidity

## Abstract

**Background:**

Temporomandibular disorders (TMDs) are common and cause persistent pain. Comorbidities are associated with TMDs and can affect the effectiveness of their treatments. The literature is lacking enough evidence on the difference between acute and chronic pain, particularly in TMDs. Investigating this difference could highlight potential risk factors for the transition from acute to chronic pain–related TMDs.

**Aim:**

To compare the likelihood of back and neck pain (BP, NP) between acute and chronic pain–related TMDs (AP-TMD, CP-TMD) as defined by pain duration and pain-related disability.‎

**Methods:**

Participants with AP-TMDs (≤3 months) and CP-TMDs (>3 months) were recruited according to the diagnostic criteria and research diagnostic criteria of TMD. BP and NP were assessed using a self-reported checklist. CP-TMDs defined by disability (chronic disability) and depression and anxiety symptoms were assessed using validated instruments. Logistic regression analyses were employed.

**Results:**

This study enrolled 487 adults with AP-TMD (*n* = 118) and CP-TMD (*n* = 369). Relative to AP-TMD, participants with CP-TMD had twice the odds of reporting NP (odds ratio [OR] = 2.17‎, 95% CI 1.27–3.71) but not BP ‎‎(OR = 0.96, 95% CI 0.57–1.64). Participants with chronic disability were twice as likely to report NP ‎(OR = 1.95‎, 95% CI 1.20–3.17‎) but not BP (OR = 1.13, 95% CI 0.69–1.82)‎ compared to those without. All analyses were adjusted for age, sex, and anxiety and depression symptoms.

**Conclusions:**

Within the limitations of this study, results suggest that central dysregulation or trigeminocervical convergence mechanisms are implicated in the process of pain-related TMD chronification and highlight the relevance of considering disability when defining CP-TMDs.

## RESUMEN

**Contexte** : Les troubles temporo-mandibulaires (TTM) sont fréquents et provoquent des douleurs persistantes. Des comorbidités sont associées aux TTM et peuvent affecter l'efficacité de leur traitement. Il n'y a pas suffisamment de données probantes dans la litt\érature sur la différence entre la douleur aiguë et la douleur chronique, en particulier dans les TTM. L'étude de cette différence pourrait mettre en évidence les facteurs de risque potentiels pour la transition des TTM liés à la douleur aiguë aux TTM liés à la douleur chronique.**Objectif** : Comparer la probabilité de douleur au dos et de douleur cervicale (DD, DC) entre les TTM liés à la douleur aiguë et les TTM liés à la douleur chronique (TTM-DA, TTM-DC), telles que définis par la durée de la douleur et l'incapacité liée à la douleur.**Méthodes** : Les participants atteints de TTM-DA (≤3 mois) et de TTM-DC (>3 mois) ont été recrutés selon les critères diagnostiques et les critères diagnostiques pour la recherche en matière de TTM. La douleur au dos et la douleur cervicale ont été évaluées à l'aide d'une liste de contrôle autodéclarée. Les TTM-DC définis par l'invalidité (invalidité chronique) et les symptômes de dépression et d'anxiété ont été évalués à l'aide d'instruments validés. Des analyses de régression logistique ont été utilisées.**Résultats** : Cette étude a inclus 487 adultes atteints de TTM-DA (n = 118) et de TTM-DC (n = 369). Comparativement aux patients atteints de TTM-DA, les participants atteints de TTM-DC étaient deux fois plus susceptibles de déclarer de la douleur cervicale (rapport de cotes [RC] = 2,17, IC à 95 % 1,27-3,71) mais pas de douleur au dos (RC = 0,96, IC à 95 % 0,57-1,64). Les participants ayant une incapacité chronique étaient deux fois plus susceptibles de déclarer de la douleur cervicale (RC = 1,95, IC à 95 % 1,20-3,17), mais pas de douleur au dos (RC = 1,13, IC à 95 % 0,69-1,82) que ceux n'en ayant pas. Toutes les analyses ont été ajustées en fonction de l'âge, du sexe, de l'anxiété et des symptômes de dépression.**Conclusions** : Dans les limites de cette étude, les résultats indiquent que la dysrégulation centrale ou les mécanismes de convergence trigéminocervicale sont impliqués dans le processus de chronicisation des TTM liés à la douleur et soulignent la pertinence de tenir compte de l’incapacité au moment de définir les TTM-DC.

## Introduction

Temporomandibular disorders (TMDs) are a group of orofacial pain conditions that affect 5% to 12% of the population.^1^ This term is used to refer to an array of painful disorders affecting the masticatory muscles (e.g., myofascial pain) and/or the temporomandibular joint (e.g., arthralgia) or the surrounding structures.^2^ TMDs present a heavy burden on the health care system and economy^3–5^ and have negative impacts on patients’ quality of life and functioning.^6–8^ Despite the wide range of treatments proposed to manage TMDs,^9^ pain usually persists or worsens in almost one-third of patients.^10^

Painful comorbidities are very common with TMDs.^11^ Randomized clinical trials demonstrated that comorbidities affected the effectiveness of the treatment tested.^12,13^ These coexisting conditions, particularly back and neck pain, are not only highly associated with chronic pain–related TMDs^11,14–16^ but also increase the risk of its development.^17,18^ There are several hypotheses attempting to explain these associations, including neuronal convergence, ‎central sensitization, and inhibition of the descending pain downregulation mechanisms.^19^

A recent critical review found a few differences between acute and chronic TMD.^20^ Nguyen et al. found that coexisting pain beyond orofacial areas (e.g., facial pain, neck, abdomen) was more common among patients with chronic pain–related TMDs and only participants with chronic pain–related TMDs presented certain comorbidities (e.g., fibromyalgia, chronic fatigue syndrome).^21^ A borderline difference was found with disability score (*P* = 0.07) between the two TMD pain groups.^22^ Due to methodological weaknesses in the available literature, more research is required to establish the differences between acute and chronic pain–related TMDs. This differentiation is very relevant because it may indicate new potential factors associated with the transition from acute to chronic pain.

Therefore, the present study aimed to compare the likelihood of back pain and neck pain between acute and chronic pain–related TMDs as defined by pain duration (≤3 months versus >3 months) and pain-related disability (high disability versus low disability).

## Materials and Methods

### Overview

The current case–control analysis comparing the acute and the chronic cohorts of the ACTION project is described below. The ACTION project is a multisite prospective cohort study investigating the risk factors for the transition of acute to chronic pain–related TMDs as well as its persistence.

The ACTION project was approved by the McGill Institutional Review Board in Montreal (Approval No. A12-M113-14A) and by the Dental Specialists Group in Ottawa (Approval No. 240–400) and complied with the Declaration of Helsinki. All participants agreed to participate in this study and signed a consent form. Written signatures were obtained until December 2021 and online signatures were obtained after December 2021.

### Study Population

Participants of the current study were recruited from the ACTION cohort between August 2015 and March 2021‎ from four sites: (1) the Jewish General Hospital general dental clinic, (2) the Faculty of Dentistry of McGill ‎University oral diagnosis clinic, (3) Montreal General Hospital, and (4) the Dental Specialists ‎Group TMD-specialized clinic.

All potential participants presenting to the recruitment sites were considered for enrollment and were invited to ‎participate. To be included in the study, patients had to be diagnosed with pain-related TMDs ‎(muscle and/or joint pain) ‎in accordance with the diagnostic criteria (DC) and research diagnostic criteria (RDC) of TMD and be between 18 and 85 years of age. These protocols have been proven to have high validity and reliability particularly for pain-related TMDs.^23,24^ Patients with other orofacial pain or cancer were excluded in order to decrease the likelihood of information bias. Patients without a telephone or who were unable to understand English or French or provide informed consent were excluded. ‎

### Classification of Acute and Chronic Pain–related TMDs

#### Classification of Pain-related TMDs Based on Pain Duration

When defined by pain duration, pain-related TMD was classified according to the ‎recent chronic pain definition by the International Association for the Study of Pain (IASP): ‎‎“Pain that lasts or recurs for longer than 3 months.”^25^ Therefore, the outcome was chronic pain–related TMDs (pain for more than 3 months), and the control group was acute pain (pain lasting for 3 ‎months or less). This also agrees with the International Classification of Orofacial Pain.^26^

#### Classification of Pain-related TMDs Based on Pain-related Disability

Based on the recommendations by the IASP that considered disability as a significant factor associated with chronic pain,^25^ we classified pain-related TMDs based on disability using the Graded ‎Chronic Pain Scale (GCPS). The GCPS ‎evaluates pain-related disability hierarchically, with more ‎disability expressed as a higher ‎grade. According to the GCPS, disability is graded by its impact ‎on activities, unemployment, ‎health care utilization, medications, depression, and self-perceived health status. This scale comprises four ‎grades: Grade I: low disability, low pain intensity ‎‎(<50%); Grade II: low disability, high pain ‎intensity (≥50%); Grade III: high disability, ‎moderately limiting; Grade IV: high disability, ‎severely limiting.‎^27^ The outcome was chronic pain–related TMDs defined by disability (Grades III and IV), and the control group was non-chronic disability (Grades I and II).‎

### Assessment

#### Assessment of Back and Neck Pain

Both back and neck pain were screened using a self-reported checklist. Participants were asked whether they had these conditions and they had two choices: “Yes” or “No.”

#### Assessment of Potential Confounders and Effect Modifiers

Age, sex, anxiety, and depression symptoms were considered potential confounders. The Generalized Anxiety Disorder 7 (GAD-7) and Patient Health Questionnaire 8 (PHQ-8) were used to assess anxiety and depression symptoms, respectively. Both questionnaires have several statements to which participants respond with a score from 0 to 3 according to the frequency these statements apply to them ‎(i.e., *not at all, several ‎days, more than ‎half the ‎days, nearly ‎every ‎day*). The total scores of ‎GAD-7 and PHQ-8 are 21 and 24, respectively. These instruments were proven to have high ‎specificity and sensitivity, with scores of 5, 10, and 15 referring to mild, moderate, and severe ‎anxiety or depression, respectively.^28–31^ In our study, a cutoff of 5 was used to detect the presence of anxiety or depression symptoms. In addition, age and sex were included as sociodemographic variables.

### Statistical Analysis

Student’s *t* and ‎chi-square tests were used to assess statistical differences between participants with acute and ‎chronic pain–related TMDs for continuous and categorical variables, respectively.‎

Univariate and multivariable unconditional bivariate logistic regression models were ‎employed to compare the odds of neck and back pain between acute and chronic pain–related TMDs as defined by pain duration (aim 1) and pain-related disability (aim 2). The multivariable logistic models also included age, sex, and anxiety and ‎depression symptoms as potential confounders or effect modifiers.

The odds ratios (OR) and 95% confidence intervals (95% CIs) were estimated. ‎SAS statistical software (v9.4) was used to perform the analyses with the ‎‎significance level for type I error ‎set at 0.05.‎ Considering the sample size used (‎369 patients with chronic pain–related TMD and 118 with acute pain‎) and the prevalence of back (64%) and neck pain (55%) among the group with chronic pain,^11^ this study had a statistical power of 83% and 88% to detect an OR as low as 2.0 for back and neck pain, respectively.

## Results

### Description of the Sample

**Figure 1.**
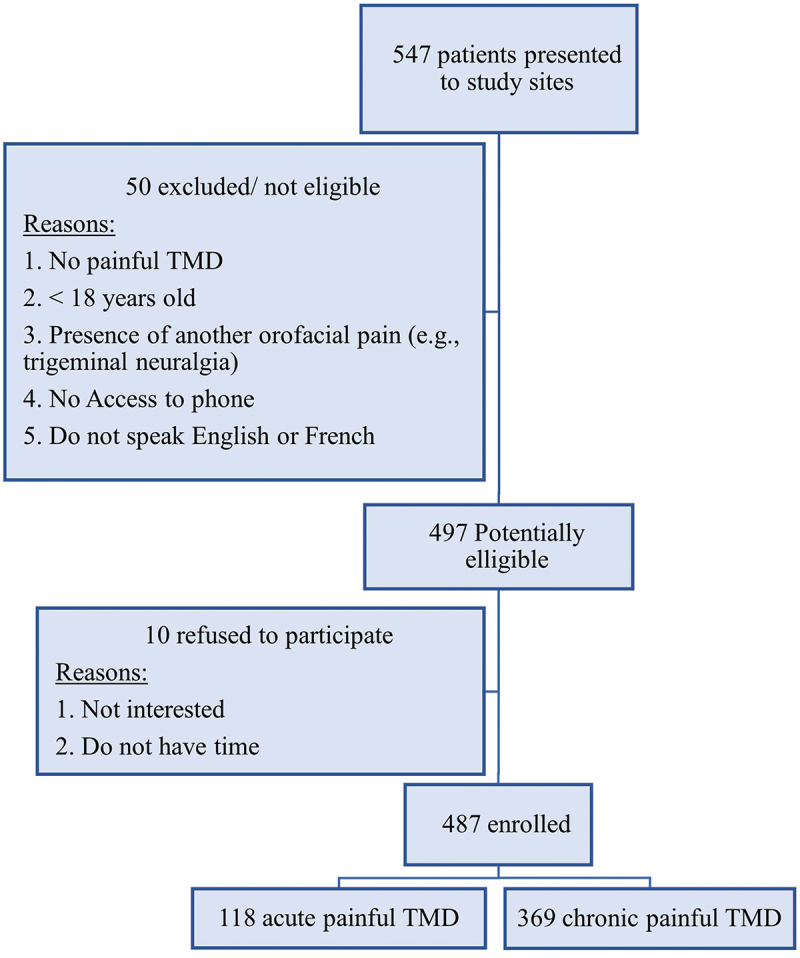
Flowchart showing the recruitment‎ process.

Table 1 compares the characteristics of the sample between the acute and chronic pain–related TMD groups. Out of 547 potential participants at the study sites, 487 were enrolled in this study (89.03%). Further details about the recruitment process are presented in Figure 1. Among those included, 118 (24.22%) had acute pain–related TMDs and 369 (75.77%) had chronic pain defined by pain duration. The chronic pain–related TMDs group had a significantly higher number of females ‎(*P* = ‎0.038)‎ than the group with acute pain, and the mean age was similar between both groups.‎ Participants recruited from Ottawa represented ‎23% (*n* = 112) of the total sample, and those recruited from Montreal accounted for 77% (*n* = 375). This is due to the presence of three recruitment sites in Montreal and one in Ottawa. However, the distribution of participants from the two cities in the study groups was not significantly different (*P* = 0.22).

**Table 1.** Comparison between acute and chronic pain–related TMD groups based on the sample characteristics.

	Category	Acute painful TMD^a^ (*n* = 118)	Chronic painful TMD^b^ (*n* = 369)	*P* value	Missing data, *n* (%)
Back pain, *n* (%)	No Yes	75 (64.10) 42 (35.90)	190 (51.49) 179 (48.51)	‎‎0.017‎‎	‎20 (4.1)‎
Neck pain, *n* (%)‎	No Yes	81 (68.64) 37 (31.36)	182 (49.49) 185 (50.41)	<0.001	21 (4.3)
Age	Mean (SD)	42.82 (16.63)	42.36 (16.35)	0.79	‎19 (3.9)‎
Sex, *n* (%)‎	Male Female	37 (31.36) 81 (68.64)	81 (21.95) 288 (78.05)	0.038	19 (3.9)
City, *n* (%)‎	Montreal Ottawa	86 (72.88) 32 (27.12)	289 (78.32) 80 (21.68)	0.22	19 (3.9)
Graded Chronic Pain Scale, *n* (%)‎	Grade I Grade II Grade III Grade IV	33 (27.97) 35 (29.66) 21 (17.80) 29 (24.58)	110 (29.81) 133 (36.05) 54 (14.63) 72 (19.51)	0.34	19 (3.9)
Anxiety, *n* (%)‎	No Yes^c^	50 (42.74) 67 (57.26)	130 (35.23) 239 (64.77)	0.14	‎20 (4.1)‎
Depression, *n* (%)‎	No Yes^d^	41 (35.04) 76 (64.96)	73 (19.78) 296 (80.22)	<0.001	20 (4.1)

^a^≤3 months.

^b^>3 months.

^c^GAD-7 score ≥5.

^d^PHQ-8 score ≥5.

### Chronic TMD Defined by Pain Duration

Self-reported back (*P* = ‎0.017‎) and neck (*P* < 0.001) pain were more frequent among patients with chronic than acute pain–related TMDs‎. More than one-third of the group with chronic pain was classified as Grade II according to the GCPS (*n* = ‎133, 36.05%)‎. Depression symptoms were common in both groups but significantly more prevalent among the chronic pain–related TMD‎s group relative to the group with acute pain (64.96%, *P* < 0.001)‎. Anxiety, however, was not different between the two groups. The percentage of missing data was 4%.

Table 2 demonstrates the association of back and neck pain with chronic compared to ‎acute pain–related TMDs based on pain duration (≤3 months versus >3 months). Participants with chronic pain–related TMDs had twice the likelihood of reporting neck pain ‎(OR = ‎2.17‎, 95% CI ‎1.27–3.71‎‎) but not back pain ‎(OR = ‎0.96, 95% CI ‎0.57–1.64‎)‎ compared to those with acute pain–related TMDs, regardless of age, sex or anxiety, and depression symptoms‎. Furthermore, depression symptoms ‎(OR = ‎2.08‎, ‎95% CI‎ 1.21–3.58‎) ‎were associated with chronic pain–related TMDs, whereas age, sex, and anxiety symptoms did not show statistically significant ORs.

**Table 2.** The association between neck and back pain and chronic^a^ relative to acute^b^ pain–related TMDs defined by pain duration.

		Crude		Adjusted^c^	
	Category	Odds ratios	95% CI	OR	95% CI
Back pain	No Yes	1 1.68*	Reference 1.10–2.58	1 0.96	Reference 0.57–1.64
Neck pain	No Yes	1 2.23*	Reference 1.43–3.45	1 2.17*	Reference 1.27–3.71
Age		1.05	0.66–1.67	1.00	0.99–1.01
Sex	Male Female	1 1.62*	Reference 1.03–2.57	1 1.51	Reference 0.93–2.44
Anxiety	No Yes^d^	1 ‎1.26 ‎	Reference ‎0.83–1.92	1 0.97	Reference 0.59–1.60
Depression	No Yes^e^	1 2.19*	Reference 1.38–3.46	1 2.08*	Reference 1.21–3.58

^a^>3 months.

^b^≤3 months.

^c^Adjusted for age, sex, and anxiety and depression symptoms,

^d^GAD-7 score ≥5,

^e^PHQ-8 score ‎≥5,

*P < 0.05.

### Chronic Disability TMDs

Table 3 shows the crude and adjusted ORs of the logistic regression analysis assessing the ‎association of neck and back pain comorbidities with chronic pain–related TMDs defined by disability (GCPS ‎Grades III–IV). Similar to the results presented in Table 2, participants with chronic disability ‎were almost twice as likely to report neck pain ‎(OR = ‎1.95‎, 95% CI ‎1.20–3.17) ‎compared to those with non-chronic pain–related disability, regardless of participant age, sex, anxiety and depression symptoms, presence of back pain, ‎and the acute/chronic pain status defined by pain duration. The covariates associated with the study ‎outcome were anxiety (‎OR = ‎‎‎2.43‎‎, 95% CI ‎1.51–3.90‎), depression symptoms‎ (OR = ‎‎‎‎1.85‎‎‎, 95% CI ‎‎1.04–3.29‎)‎, and acute–chronic pain status defined by pain duration (OR = ‎‎‎0.51‎‎, 95% CI ‎0.32–‎‎0.81‎‎‎)‎. Conversely, participants in the group with chronic disability did not show an increased ‎likelihood of reporting back pain ‎(OR = ‎‎1.13‎, 95% CI ‎‎0.69–1.82) compared to those in the non-chronic disability group.

**Table 3.** The association between neck and back pain and chronic^a^ relative to non-chronic^b^ pain–related TMDs defined by pain-related disability.

		Crude		Adjusted ^c^	
	Category	OR	95% CI	OR	95% CI
Back pain	No Yes	1 1.59*	Reference 1.09–2.30	1 1.13	Reference 0.69–1.82
Neck pain	No Yes	1 1.82*	Reference 1.25–2.65	1 1.95*	Reference 1.20–3.17
Age		0.99	0.98–1.00	0.99	0.98–1.00
Sex	Male Female	1 0.87	Reference 0.57–1.32	1 0.82	Reference 0.52–1.30
Anxiety	No Yes^d^	1 3.29*	Reference 2.22–4.88	1 2.43*	Reference 1.51–3.90
Depression	No Yes^e^	1 2.51*	Reference 1.59–3.97	1 1.85*	Reference 1.04–3.29
Acute/chronic pain–related TMD status	Acute^f^ Chronic^g^	1 0.75	Reference 0.50–1.13	1 0.51*	Reference 0.32–0.81

^a^GCPS Grades III–IV.

^b^GCPS Grades I–II.

^c^Adjusted for age, sex, anxiety and depression symptoms, and acute/chronic painful TMD status.

^d^GAD-7 score ≥5.

^e^PHQ-8 score ‎≥5.

^f^>3 months.

^g^≤3 months.

*P < 0.05.

## Discussion

Several studies in the literature have demonstrated that individuals with chronic pain–related TMDs report back or neck pain more frequently than those without.^11,14–16^ Additionally, both comorbidities increased the risk of pain-related TMDs.^17,18^ However, to the best of our knowledge, this is the first study comparing the likelihood of back and neck pain between acute and chronic pain–related TMDs.

Studies similar to ours are scarce in the literature. One cohort study assessed the association between pain-related TMD duration, widespread pain, and painful comorbidities including back and neck pain. In that study, results showed that increased pain-related TMD duration was associated with an increased odds of having painful comorbidities as well as pain beyond the orofacial region.^32^ In our study, participants with chronic pain–related TMDs were twice as likely to report neck pain relative to those with acute pain. Similarly, other studies found that the presence of painful comorbidities increased the risk of chronic postoperative pain and the odds of the transition from acute to chronic postsurgical pain.^33,34^

Kotiranta ‎and colleagues assessed the relationship between pain-related disability in patients with TMD diagnosed according to RDC/TMD and comorbid pain conditions. This study found that participants with TMD with high disability (three to six points on the GCPS) presented a greater number of comorbidities (e.g., headache, back pain, neck pain, abdominal pain) relative to the nondisabled group (zero points).^35^ In our current study, participants with GCPS Grades III–IV (i.e., high disability) had double the odds of neck pain compared to those with low disability (Grades I–II).

Interestingly, highly disabled participants had acute rather than chronic pain–related TMDs. This might be related to the treatment-seeking behavior of these acute cases and ‎cannot be generalized. Participants with acute pain–related TMDs who sought medical care reported ‎high levels of disability more frequently than the group with chronic pain. This agrees with a previous ‎study that showed that treatment seekers were more likely to have a shorter duration of pain-related TMD and a higher disability score.^36^ One reason for this association could be that patients with pain-related TMDs‎ with high disability usually seek care as early as possible in order to receive treatment. ‎On the contrary, those without disability wait until the pain has persisted past the ‎acute–chronic threshold (i.e., 3 months).‎

Our findings suggest that central dysregulation mechanisms^37,38^ are implicated in the process of pain-related TMD chronification involving peripheral and central sensitization mechanisms. Central pain is characterized as being diffuse or multifocal and thus is associated with comorbid pain conditions.^19,39^ Another suggested mechanism is trigeminocervical convergence.^40–42^ The neurons in the trigeminal nucleus caudalis that extend to C2 and the lateral cervical nucleus are stimulated by trigeminal activation, causing symptoms in both the trigeminal and cervical regions. This mechanism could be activated as pain-related TMDs becomes chronic, leading to the observed association between chronic pain–related TMDs and neck pain but not back pain. Moreover, the association of chronic disability with neck pain calls attention to the importance of including disability as a factor defining chronic pain–related TMDs in addition to pain duration, which agrees with the latest IASP recommendations.^25^ This accurate distinction will aid clinicians in developing the most suitable and effective management protocols, which may involve a multidisciplinary team to address comorbidities associated with pain persistence or disability.

On the other hand, it is possible that back pain is more frequently reported in specific subgroups or subdiagnoses (e.g., myofascial pain) of chronic pain–related TMDs compared to acute pain. Several studies proposed that different mechanisms are implicated in these subgroups or subdiagnoses,^43–45^ thus leading to different associations with comorbidities.

The limitations of this study should be noted. First, temporality cannot be established in case–control studies. Due to the study design, whether exposures precede outcomes cannot be ascertained. Therefore, no causal relationships can be inferred. Second, self-report questionnaires were used in this study to evaluate anxiety and depression symptoms, back and neck pain, and disability. Though reliable and validated, these self-report questionnaires are liable to have recall and misclassification biases. However, the estimated prevalence of back and neck pain among participants with chronic pain–related TMD in the current study was lower than that reported by Plesh et al.^11^ This suggests that the estimates provided in our study were under- rather than overestimated. Third, subanalyses to assess sex and site differences were not possible due to insufficient sample sizes in the respective subgroups. Fourth, variables such as ethnicity, socioeconomic status, and education level were not collected, and TMD subdiagnoses were not included in the analysis; thus, it was not possible to assess their potential confounding effects. Additionally, only the presence or absence of back and neck pain was assessed. Other determinants of back and neck pain such as the duration or frequency of pain were not included.

Strengths of this study include the following: this study was a multisite study conducted in four sites across two cities in two different provinces. The recruitment of participants at different sites not only reduces the chance of selection bias but also improves the external validity of the study. Moreover, this did not introduce any selection bias because the percentage of participants recruited from Montreal and Ottawa was similar in both study groups, as shown in Table 1. Second, we used highly valid clinical instruments (DC and RDC/TMD) to diagnose participants in both groups. Anxiety and depression symptoms and disability were also assessed with validated and reliable questionnaires. In addition, the most updated IASP definition of chronic pain using a 3-month threshold was used to classify acute and chronic pain–related TMDs based on pain duration.^25^ This reduces the chance of misclassification bias and enhances the validity of our results. Third, an analysis was conducted to compare the likelihood of back and neck pain when defining chronic pain–related TMDs based on disability, in accordance with the latest IASP recommendations.^25^ This analysis yielded very interesting results.

In conclusion, we found that participants with chronic pain–related TMD had an increased likelihood of reporting neck pain but not back pain when compared to the group with acutepain . These results were maintained when chronic pain–related TMDs were defined by high disability. This suggests some potential risk factors for the transition from acute to chronic pain–related TMDs that can be investigated in future research and highlights possible mechanisms for this transition. In addition, it sheds light on the relevance of identifying disability in patients with chronic pain.

## Supplementary Material

Supplemental Material
